# Surveillance To Track Progress Toward Poliomyelitis Eradication — Worldwide, 2021–2022

**DOI:** 10.15585/mmwr.mm7223a1

**Published:** 2023-06-09

**Authors:** Tasha Stehling-Ariza, Amanda L. Wilkinson, Ousmane M. Diop, Jaume Jorba, Humayun Asghar, Tigran Avagnan, Varja Grabovac, Ticha Johnson, Sudhir Joshi, Anfumbom K. W. Kfutwah, Lucky Sangal, Salmaan Sharif, Ashraf Wahdan, Graham F. Tallis, Stephanie D. Kovacs

**Affiliations:** ^1^World Health Organization, Geneva, Switzerland; ^2^Global Immunization Division, Center for Global Health, CDC; ^3^Division of Viral Diseases, National Center for Immunization and Respiratory Diseases, CDC; ^4^World Health Organization Regional Office for the Eastern Mediterranean, Amman, Jordan; ^5^World Health Organization Regional Office for the Western Pacific, Manila, Philippines; ^6^World Health Organization Regional Office for Africa, Brazzaville, Republic of the Congo; ^7^World Health Organization Regional Office for South-East Asia, New Delhi, India.

Since the Global Polio Eradication Initiative (GPEI) was established in 1988, the number of wild poliovirus (WPV) cases has declined by >99.9%, and WPV serotypes 2 and 3 have been declared eradicated (*1*). By the end of 2022, WPV type 1 (WPV1) transmission remained endemic only in Afghanistan and Pakistan (*2*,*3*). However, during 2021–2022, Malawi and Mozambique reported nine WPV1 cases that were genetically linked to Pakistan (*4*,*5*), and circulating vaccine-derived poliovirus (cVDPV) outbreaks were detected in 42 countries (*6*). cVDPVs are oral poliovirus vaccine-derived viruses that can emerge after prolonged circulation in populations with low immunity allowing reversion to neurovirulence and can cause paralysis. Polioviruses are detected primarily through surveillance for acute flaccid paralysis (AFP), and poliovirus is confirmed through stool specimen testing. Environmental surveillance, the systematic sampling of sewage and testing for the presence of poliovirus, supplements AFP surveillance. Both surveillance systems were affected by the COVID-19 pandemic’s effects on public health activities during 2020 (*7*,*8*) but improved in 2021 (*9*). This report updates previous reports (*7*,*9*) to describe surveillance performance during 2021–2022 in 34 priority countries.* In 2022, a total of 26 (76.5%) priority countries met the two key AFP surveillance performance indicator targets nationally compared with 24 (70.6%) countries in 2021; however, substantial gaps remain in subnational areas. Environmental surveillance expanded to 725 sites in priority countries, a 31.1% increase from the 553 sites reported in 2021. High-quality surveillance is critical to rapidly detect poliovirus transmission and enable prompt poliovirus outbreak response to stop circulation. Frequent monitoring of surveillance guides improvements to achieve progress toward polio eradication.

## Acute Flaccid Paralysis Surveillance

**Performance Indicators.** AFP surveillance quality is assessed using two performance indicators: 1) the nonpolio AFP (NPAFP) rate, by which an NPAFP rate of at least two per 100,000 persons aged <15 years is deemed sufficiently sensitive to detect circulating poliovirus, and 2) stool adequacy,^†^ with the target of ≥80% adequate stool specimens collected from AFP patients indicating effective identification of poliovirus. Surveillance indicators were assessed in 34 priority countries during 2021–2022 and summarized by region (Table 1).

**TABLE 1:** National and subnational acute flaccid paralysis surveillance performance indicators and number of confirmed wild poliovirus and circulating vaccine-derived poliovirus cases, by country — 34 priority countries, World Health Organization African, Eastern Mediterranean, South-East Asia, and Western Pacific Regions, 2021–2022*

Year/WHO region/Country	No. of AFP cases	Regional or national NPAFP rate^†^	Percentage				No. of confirmed cases	
			Subnational areas with NPAFP rate ≥2^§^	Regional or national AFP cases with adequate specimens^¶^	Subnational areas with ≥80% adequate specimens^¶^	Population living in areas meeting both indicators**	WPV	cVDPV (type 1, type 2)^††^
**2021**								
**African **	**23,345**	**5.9**	**NA**	**88.9**	**NA**	**NA**	**1**	**512 (13, 499)**
Angola	471	2.9	88.9	83.0	66.7	46.7	—^§§^	—
Benin	259	4.9	100.0	88.4	91.7	97.0	—	3 (0, 3)
Burkina Faso	1,400	14.5	100.0	90.2	100.0	100.0	—	2 (0, 2)
Cameroon	755	6.7	100.0	82.9	50.0	43.7	—	3 (0, 3)
Central African Republic	202	8.9	100.0	77.2	28.6	35.1	—	—
Chad	1,055	13.6	100.0	84.6	69.6	70.3	—	—
Côte d’Ivoire	738	6.6	100.0	84.8	75.8	81.9	—	—
Democratic Republic of the Congo	3,444	7.9	100.0	85.3	84.6	91.0	—	28 (0, 28)
Equatorial Guinea	19	3.6	42.9	89.5	57.1	24.0	—	—
Ethiopia	1,694	3.7	90.9	91.5	100.0	94.6	—	10 (0, 10)
Guinea	370	6.2	100.0	79.5	50.0	49.6	—	6 (0, 6)
Guinea-Bissau	20	1.9	36.4	65.0	27.3	28.3	—	3 (0, 3)
Kenya	660	3.0	80.9	85.9	68.1	55.9	—	—
Madagascar	602	5.1	100.0	94.7	100.0	100.0	—	13 (13, 0)
Malawi	177	1.9	50.0	75.1	50.0	54.8	1	—
Mali	448	4.6	100.0	84.6	81.8	80.7	—	—
Mozambique	468	3.1	100.0	73.7	27.3	19.2	—	2 (0, 2)
Niger	628	4.9	100.0	83.4	75.0	75.0	—	18 (0, 18)
Nigeria	7,801	8.0	100.0	93.9	100.0	100.0	—	415 (0, 415)
South Sudan	565	9.1	100.0	89.2	100.0	100.0	—	9 (0, 9)
Tanzania	885	3.1	90.3	97.9	96.8	90.9	—	—
Togo	298	8.6	100.0	92.6	100.0	100.0	—	—
Zambia	244	2.8	100.0	65.6	10.0	15.1	—	—
Zimbabwe	142	1.5	50.0	84.5	80.0	52.1	—	—
**Eastern Mediterranean **	**20,261**	**13.5**	**NA**	**87.1**	**NA**	**NA**	**5**	**121 (3, 118)**
Afghanistan	4,095	25.5	100.0	93.4	100.0	100.0	4	43 (0, 43)
Iraq	709	4.2	94.7	91.1	94.7	85.5	—	—
Pakistan	13,119	18.1	85.7	84.8	100.0	100.0	1	8 (0, 8)
Somalia	349	4.6	90.5	96.0	90.5	83.0	—	1 (0, 1)
Sudan	637	3.6	100.0	94.0	100.0	100.0	—	—
Syria	431	6.7	92.9	85.4	78.6	61.9	—	—
Yemen	921	7.1	100.0	81.7	78.3	67.0	—	69 (3, 66)
**South-East Asia **	**33**	**0.2**	**NA**	**84.8**	**NA**	**NA**	—	—
Burma (Myanmar)^¶¶^	33	0.2	0	84.8	33.3	0	—	—
**Western Pacific **	**946**	**2.5**	**NA**	**76.8**	**NA**	**NA**	—	—
Papua New Guinea	52	1.3	13.6	50.0	13.6	0	—	—
Philippines	894	2.6	82.4	78.4	52.9	43.9	—	—
**2022**								
**African**	**29,024**	**7.2**	**NA**	**90.2**	**NA**	**NA**	**8**	**653 (173, 480)**
Angola	384	2.4	66.7	89.3	77.8	74.2	—	—
Benin	337	6.1	100.0	82.5	75.0	69.2	—	11 (0, 11)
Burkina Faso	1,250	12.7	100.0	93.0	100.0	100.0	—	—
Cameroon	852	7.4	100.0	81.9	60.0	64.7	—	3 (0, 3)
Central African Republic	215	9.6	100.0	86.0	57.1	64.9	—	5 (0, 5)
Chad	1,254	15.2	95.7	82.1	52.2	53.7	—	44 (0, 44)
Côte d’Ivoire	788	6.8	100.0	81.1	66.7	62.6	—	—
Democratic Republic of the Congo	4,561	9.2	100.0	85.9	61.5	61.2	—	478 (133, 345)
Equatorial Guinea	20	3.5	57.1	75.0	57.1	26.3	—	—
Ethiopia	1,606	3.5	90.9	93.0	90.9	92.7	—	1 (0, 1)
Guinea	389	6.5	100.0	86.6	87.5	85.2	—	—
Guinea-Bissau	38	3.7	72.7	52.6	36.4	15.7	—	—
Kenya	634	2.9	83.0	86.3	74.5	71.0	—	—
Madagascar	647	5.4	100.0	95.1	100.0	100.0	—	14 (14, 0)
Malawi	470	4.8	100.0	71.3	25.0	0.1	—	4 (4, 0)
Mali	562	5.6	100.0	87.4	90.9	99.5	—	2 (0, 2)
Mozambique	928	5.7	90.9	74.6	27.3	15.7	8	26 (22, 4)
Niger	990	7.5	100.0	87.8	75.0	75.0	—	15 (0, 15)
Nigeria	10,247	10.9	100.0	96.7	100.0	100.0	—	48 (0, 48)
South Sudan	557	9.5	100.0	94.3	100.0	100.0	—	—
Tanzania	1,286	4.4	93.5	98.1	100.0	98.6	—	—
Togo	277	7.8	100.0	89.5	100.0	100.0	—	2 (0, 2)
Zambia	382	4.2	100.0	66.8	10.0	18.7	—	—
Zimbabwe	350	4.6	100.0	88.3	90.0	90.6	—	—
**Eastern Mediterranean**	**27,993**	**18.5**	**NA**	**87.3**	**NA**	**NA**	**22**	**168 (0, 168)**
Afghanistan	5,370	33.7	100.0	94.4	100.0	100.0	2	—
Iraq	835	4.8	100.0	91.7	94.7	87.6	—	—
Pakistan	19,023	26.0	85.7	84.9	100.0	100.0	20	—
Somalia	356	4.4	90.5	97.2	95.2	96.7	—	5 (0, 5)
Sudan	650	3.7	100.0	97.1	94.4	98.1	—	1 (0, 1)
Syria	382	5.8	92.9	91.4	92.9	85.2	—	—
Yemen	1,377	10.1	100.0	81.0	60.9	59.7	—	162 (0, 162)
**South-East Asia**	**150**	**1.1**	**NA**	**88.0**	**NA**	**NA**	—	—
Burma (Myanmar)^¶¶^	150	1.1	16.7	88.0	61.1	11.7	—	—
**Western Pacific**	**816**	**2.1**	**NA**	**77.6**	**NA**	**NA**	—	—
Papua New Guinea	64	1.4	9.1	57.8	27.3	4.0	—	—
Philippines	752	2.2	29.4	79.3	35.3	11.0	—	—

**Abbreviations:** AFP = acute flaccid paralysis; cVDPV = circulating vaccine-derived poliovirus; NA = not applicable; NPAFP = nonpolio acute flaccid paralysis; WHO = World Health Organization; WPV = wild poliovirus.

* Data as of April 25, 2023.

^†^ Per 100,000 persons aged <15 years per year.

^§^ For all subnational areas regardless of population size.

^¶^ Standard WHO target is adequate stool specimen collection from ≥80% of AFP patients, assessed by timeliness and condition. For this analysis, timeliness was defined as two specimens collected ≥24 hours apart (≥1 calendar day in this data set), both ≤14 days of paralysis onset. Good condition was defined as arrival of specimens in a WHO-accredited laboratory via reverse cold chain (a transportation and storage method designed to keep samples at recommended temperatures from collection through arrival at the laboratory) and without leakage or desiccation.

** Percentage of the country’s population aged <15 years living in subnational areas that met both surveillance indicators (NPAFP rates two or more per 100,000 persons aged <15 years per year and ≥80% of AFP cases with adequate specimens).

^††^ https://polioeradication.org/wp-content/uploads/2016/09/Reporting-and-Classification-of-VDPVs_Aug2016_EN.pdf

^§§^ Dashes indicate that no confirmed cases were found.

^¶¶^ *MMWR* uses the U.S. Department of State’s short-form name “Burma”; WHO uses “Myanmar.”

**African Region.** Among 24 priority countries in the World Health Organization (WHO) African Region (AFR), 19 (79.2%) met both national AFP surveillance indicator targets in 2022 compared with 17 (70.8%) in 2021 (Table 1). In 2022, all 24 countries met the NPAFP rate target nationally, and 19 (79.2%) met the stool adequacy target. Indicators at the first subnational administrative level (i.e., province or state) were similar both years, with 73.2% and 72.4% of subnational areas meeting both targets in 2021 and 2022, respectively (Figure). In 2022, ≥80% of subnational areas in 21 (87.5%) countries met the NPAFP rate target compared with 20 (83.3%) countries in 2021. The stool adequacy target was met by more than 80% of subnational areas in 10 (41.7%) countries in 2022 compared with 11 (45.8%) countries in 2021. Subnational area stool adequacy performance varied; nine countries (37.5%) reported more subnational areas meeting the target in 2022, and six (25.0%) reported fewer. In 2022, eight WPV1 cases were detected, and one WPV1 case had onset in 2021. The number of VDPV cases increased from 512 (13 cVDPV type 1 [cVDPV1] cases and 499 cVDPV type 2 [cVDPV2] cases) in 2021 to 653 (173 cVDPV1 and 480 cVDPV2) in 2022.

**FIGURE:**
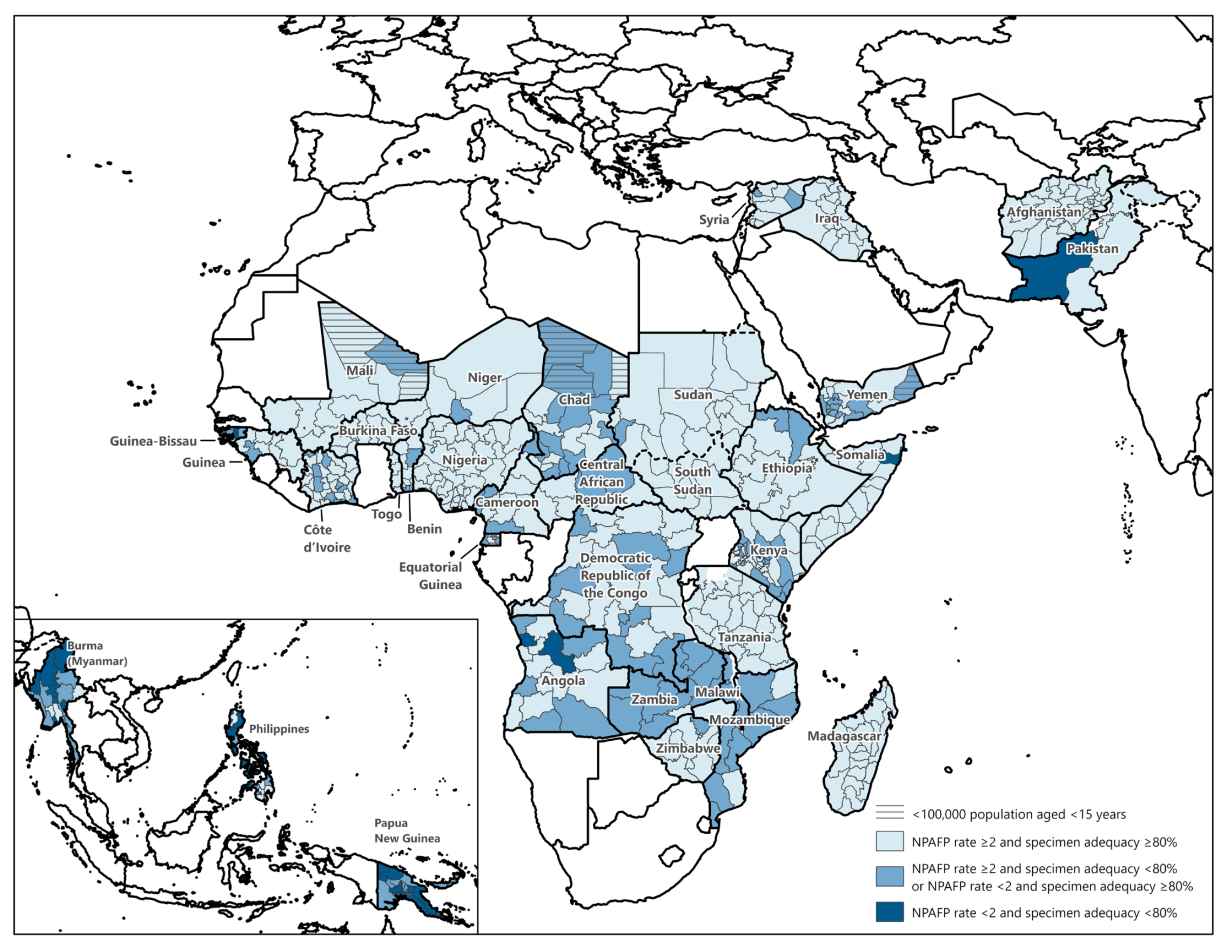
Combined performance indicators for the quality of acute flaccid paralysis surveillance* in subnational areas of 34 priority countries^†^^,^^§^ — World Health Organization African, Eastern Mediterranean, South-East Asia, and Western Pacific regions, 2022 **Abbreviations:** AFP = acute flaccid paralysis; NPAFP = nonpolio acute flaccid paralysis; WHO = World Health Organization. * Targets: Two or more NPAFP cases per 100,000 persons aged <15 years per year and ≥80% of persons with AFP having two stool specimens collected ≤14 days of paralysis onset and ≥24 hours apart and received in good condition (i.e., without leakage or desiccation) by a WHO-accredited laboratory via reverse cold chain (storing and transporting samples at recommended temperatures from the point of collection to the laboratory). ^†^ Priority countries by region in 2022 included the following: *African Region* (24): Angola, Benin, Burkina Faso, Cameroon, Central African Republic, Chad, Côte d’Ivoire, Democratic Republic of the Congo, Equatorial Guinea, Ethiopia, Guinea, Guinea-Bissau, Kenya, Madagascar, Malawi, Mali, Mozambique, Niger, Nigeria, South Sudan, Tanzania, Togo, Zambia, and Zimbabwe; *Eastern Mediterranean Region *(7): Afghanistan, Iraq, Pakistan, Somalia, Sudan, Syria, and Yemen; *South-East Asia Region *(1): Burma (Myanmar); and *Western Pacific Region* (2): Papua New Guinea and the Philippines. ^§^ NPAFP rate is difficult to interpret when the population aged <15 years is <100,000.

**Eastern Mediterranean Region.** All seven priority countries in the WHO Eastern Mediterranean Region (EMR) met both national surveillance indicator targets in 2021 and 2022. In 2022, 88.2% of subnational areas met both indicator targets compared with 89.0% in 2021. EMR reported 22 WPV1 and 168 cVDPV2 cases in 2022 compared with five WPV1, three cVDPV1, and 118 cVDPV2 in 2021.

**South-East Asia Region.** In the WHO South-East Asia Region (SEAR) priority country of Burma (Myanmar)^§^, the NPAFP rate improved from 0.2 to 1.1 cases per 100,000 but remained below the target. In 2021 and 2022, Burma met stool adequacy targets (84.8% and 88.0%, respectively). Three (16.7%) of 18 subnational areas met both NPAFP rates and stool adequacy targets in 2022 compared with none in 2021. No poliovirus cases were detected in Burma during 2021–2022.

**Western Pacific Region.** Of the two priority countries in the WHO Western Pacific Region (WPR), neither Papua New Guinea nor the Philippines met both national surveillance indicator targets during the assessment period. In Papua New Guinea, the NPAFP rate (1.3 in 2021 and 1.4 in 2022) was below target; stool adequacy increased from 50% in 2021 to 57.8% in 2022 but remained below target. In 2021 and 2022, the Philippines met NPAFP rate targets (2.6 and 2.2, respectively) but missed stool adequacy targets (78.4% and 79.3%, respectively). Across both countries, the number of subnational areas meeting both indicators declined from 20.5% in 2021 to 10.3% in 2022. No poliovirus was detected in the two WPR priority countries in 2021 or 2022.

## Environmental Surveillance

Environmental surveillance for poliovirus, the systematic collection and testing of sewage samples for poliovirus, supplements the sensitivity of AFP surveillance by detecting poliovirus circulation in the absence of confirmed paralytic polio cases. In 2022, 32 (94.1%) of the priority countries^¶^ reported 725 environmental surveillance sites compared with 553 sites in 2021, a 31.1% increase. Performance (sensitivity to detect poliovirus) is assessed by the annual enterovirus isolation rate, the proportion of environmental samples that have positive test results for any polio or nonpolio enterovirus, with a target of ≥50%.**

In AFR, the number of sites increased 11.9%, from 371 in 2021 to 415 in 2022, and the overall proportion of sites meeting the target increased from 27.8% to 40.5%. Eleven (47.8%) countries reported an increase in the proportion of sites meeting the indicator in 2022, and seven (30.4%) countries reported a decrease.

The number of sites in EMR increased from 162 in 2021 to 294 in 2022, including 120 added in Pakistan. More than 85% of sites met the indicator in each year.

In SEAR and WPR, Burma’s single reporting site met the environmental surveillance indicator. The Philippines had 17 environmental sites in 2021, five (29.4%) of which met the indicator. In 2022, 15 sites reported, and four (26.7%) met the indicator.

## Global Polio Laboratory Network

The Global Polio Laboratory Network (GPLN) consists of 144 WHO-accredited laboratories in the six WHO regions, monitored through a standardized quality assurance program of annual on-site audits and proficiency tests (*10*). All 144 GPLN laboratories are responsible for isolating polioviruses; 134 conduct intratypic differentiation to identify WPV, VDPV, and Sabin (oral poliovirus vaccine)^††^ polioviruses; and 28 conduct genomic sequencing. These 28 laboratories participated in global proficiency testing, analyzed the region of the poliovirus genome that codes for the capsid viral protein 1 (VP1), and demonstrated their ability to accurately characterize poliovirus in stool specimens (*10*).

In 2022, GPLN tested 193,945 stool specimens collected from patients with AFP (Table 2). Three of six regions (the Americas [AMR], EMR, and European [EUR]) did not meet the timeliness indicator for poliovirus isolation (results reported for ≥80% of specimens ≤14 days after receipt); however, all regions met the timeliness indicator for intratypic differentiation (results reported for more than 80% of specimens, both ≤7 days of receipt of isolate and ≤60 days of paralysis onset).

**TABLE 2:** Number of poliovirus isolates from stool specimens of persons with acute flaccid paralysis and timing of results, by World Health Organization region, 2021* and 2022**^†^**

WHO region/Year	No. of specimens	No. of poliovirus isolates			% of results		
		WPV^§^	Sabin^¶^	cVDPV**	Poliovirus isolation results ≤14 days of receipt of specimen^††,§§^	ITD results ≤7 days of receipt of isolate at laboratory^††,§§^	ITD results ≤60 days of paralysis onset^††,§§^
**African**							
2021	58,004	1	3,396	521	89	79	85
2022	53,961	8	3,065	453	86	85	83
**Americas **							
2021	1,152	0	6	0	83	100	100
2022	1,858	0	7	2	74	100	67
**Eastern Mediterranean **							
2021	43,370	5	1,050	70	97	97	94
2022	57,364	22	1,331	277	75	88	82
**European **							
2021	2,350	0	53	68	79	96	95
2022	2,980	0	22	2	79	91	91
**South-East Asia **							
2021	53,649	0	1,030	0	93	89	90
2022	67,118	0	1,067	2	96	98	93
**Western Pacific **							
2021	12,356	0	58	0	97	100	99
2022	10,664	0	32	0	98	100	100
**Total^§§^**							
**2021**	**170,881**	**6**	**5,593**	**659**	**93**	**84**	**88**
**2022**	**193,945**	**30**	**5,524**	**736**	**87**	**88**	**85**

**Abbreviations:** cVDPV = circulating vaccine-derived poliovirus; cVDPV1 = circulating vaccine-derived poliovirus type 1; cVDPV2 = circulating vaccine-derived poliovirus type 2; cVDPV3 = circulating vaccine-derived poliovirus type 3; ITD = intratypic differentiation; VDPV = vaccine-derived poliovirus; VP1 = viral protein 1; WHO = World Health Organization; WPV = wild poliovirus.

* Data as of March 10, 2022.

^†^ Data as of January 31, 2023.

^§^ Number of acute flaccid paralysis cases with WPV isolates.

^¶^ Either 1) concordant Sabin-like results in ITD test and VDPV screening, or 2) ≤1% VP1 nucleotide sequence difference compared with Sabin vaccine virus (≤0.6% for type 2).

** Includes cVDPV1, cVDPV2, and cVDPV3. For cVDPV1 and cVDPV3, 10 or more VP1 nucleotide differences from the respective poliovirus; for cVDPV2, six or more VP1 nucleotide differences from Sabin type 2 poliovirus.

^††^ Timeliness indicator targets: ≥80% of specimen results are reported within the respective time frame (poliovirus isolation ≤14 days of receipt, ITD results ≤7 days of receipt, and ITD results ≤60 days of paralysis onset).

^§§^ Total represents weighted mean percentage of regional performance.

During 2021–2022, the South Asia (SOAS) genotype was the only circulating WPV1 isolated from 36 AFP patients, 27 from the two endemic countries (Afghanistan and Pakistan), and nine from two nonendemic countries (Malawi and Mozambique). In Pakistan, 21 WPV1 cases were related to the YB3C genetic cluster (i.e., groups of polioviruses sharing ≥95% sequence identity in the region coding VP1), two of which were orphan viruses.^§§^ The YB3C cluster was also found in the nine cases from Malawi and Mozambique and two cases detected in Afghanistan. Cluster YB3A was detected in three cases in Afghanistan and in environmental samples in Pakistan. Two additional distinct clusters (YB3B and XC2B) were detected in environmental samples collected in Pakistan during 2021.

In priority countries during 2021–2022, 38 cVDPV emergence groups (eight cVDPV1 and 30 cVDPV2) were isolated from 1,454 AFP patients and 750 environmental samples. The number of cVDPV1 emergence groups increased from four isolated from 16 AFP patients and 33 environmental samples in 2021 to seven from 173 patients and 99 samples in 2022. The number of cVDPV2 emergence groups decreased from 24 isolated from 617 AFP patients and 449 environmental samples in 2021 to 17 emergence groups isolated from 648 patients and 169 samples in 2022.

## Discussion

After the COVID-19 pandemic weakened poliovirus surveillance performance (*7*,*8*), NPAFP rates during 2022 improved in most priority countries in AFR and EMR; improvements in stool adequacy, however, were only marginal. All high-priority countries in AFR and EMR met national NPAFP rate targets, and 26 of 31 countries met stool adequacy targets. Subnational surveillance gaps exist, particularly for stool adequacy, with 14 of 24 AFR countries and one of seven EMR countries reporting <80% of subnational areas meeting the target. SEAR and WPR priority countries and subnational areas showed improvement in 2022 but not enough to meet targets.

The detection of WPV1 in Malawi and Mozambique (*4*,*5*) highlights the risk for importation and the importance of monitoring surveillance performance to detect transmission. Substantial surveillance gaps persist in both countries, with <25% of the population living in areas that met both AFP surveillance indicators in 2022. The 653 VDPV cases detected in 42 countries during 2022 also emphasize the importance of sensitive and timely surveillance to help response activities interrupt poliovirus transmission. While polioviruses continue to circulate, all countries remain at risk for importation and must strengthen and maintain surveillance.

The findings in this report are subject to at least four limitations. First, the NPAFP rate depends on the accurate identification of AFP cases; however, the data presented in this study might include cases not meeting the AFP case definition and exclude actual AFP cases that were not reported. Environmental surveillance improves sensitivity without relying on AFP case detection. Second, AFP surveillance measures of timeliness depend on the accurate identification of paralysis onset date during the field investigation. Third, performance measures reported at regional and national levels can obscure variation at lower administrative levels. Finally, populations living in hard-to-access areas might not be adequately identified by the surveillance system and could affect subnational surveillance indicators and limit their interpretation. 

High-quality surveillance is critical for the timely detection of circulating poliovirus and the rapid activation of outbreak response vaccination activities to stop transmission. Countries should maintain high-quality surveillance by monitoring surveillance indicators to identify gaps, enhance the sensitivity and timeliness of surveillance activities, and guide program decision-making toward polio eradication.

**Summary**

**What is already known about this topic?**

The primary means for detecting poliovirus is through acute flaccid paralysis (AFP) surveillance, which is supplemented by environmental surveillance of sewage samples.

**What is added by this report?**

During 2021–2022, among 34 priority countries experiencing or at high risk for poliovirus transmission, 26 (76.5%) met national AFP surveillance indicator targets, and the number of environmental surveillance sites increased by 31%. However, substantial national and subnational AFP surveillance gaps persist.

**What are the implications for public health practice?**

Maintaining high-quality surveillance is critical to achieving the goal of global polio eradication. Monitoring surveillance indicators is important to identify gaps and guide surveillance-strengthening activities, particularly in countries at high risk for poliovirus circulation.

## Footnotes

* Priority countries were selected for this 2021–2022 report because of ongoing gaps in surveillance and vulnerability to poliovirus circulation, as determined in the World Health Organization Global Polio Surveillance Action Plan, 2022–2024. The plan was updated in July 2022 to reflect WPV1 detections in the African Region and increased risk for lower administration levels. Priority countries by region in 2022 included the following: *African Region* (24): Angola, Benin, Burkina Faso, Cameroon, Central African Republic, Chad, Côte d’Ivoire, Democratic Republic of the Congo, Equatorial Guinea, Ethiopia, Guinea, Guinea-Bissau, Kenya, Madagascar, Malawi, Mali, Mozambique, Niger, Nigeria, South Sudan, Tanzania, Togo, Zambia, and Zimbabwe; *Eastern Mediterranean Region* (7): Afghanistan, Iraq, Pakistan, Somalia, Sudan, Syria, and Yemen; *South-East Asia Region* (1): Burma (Myanmar); and *Western Pacific Region* (2): Papua New Guinea and the Philippines.

† Two stool specimens that are collected from AFP patients ≤14 days after paralysis onset and ≥24 hours apart and are received in good condition (i.e., without leakage or desiccation) by a WHO-accredited laboratory via reverse cold chain (a transportation and storage method designed to keep samples at recommended temperatures from collection through arrival at the laboratory).

§ *MMWR* uses the U.S. Department of State’s short-form name “Burma”; WHO uses “Myanmar.”

¶ No environmental surveillance sites were reported from Papua New Guinea or Zimbabwe during 2021–2022.

**
https://polioeradication.org/wp-content/uploads/2022/02/Global-Polio-Surveillance-Action-Plan-2022-2024.pdf

†† Sabin polioviruses are often detected during and for several weeks after vaccination campaigns that use the oral poliovirus vaccine. Detection of Sabin poliovirus does not indicate virus circulation.

§§ Orphan viruses, with ≥1.5% nucleotide divergence of the VP1-coding region from known isolates, indicate surveillance did not detect prolonged virus circulation.
